# Cross-species recognition of SARS-CoV-2 to bat ACE2

**DOI:** 10.1073/pnas.2020216118

**Published:** 2020-12-28

**Authors:** Kefang Liu, Shuguang Tan, Sheng Niu, Jia Wang, Lili Wu, Huan Sun, Yanfang Zhang, Xiaoqian Pan, Xiao Qu, Pei Du, Yumin Meng, Yunfei Jia, Qian Chen, Chuxia Deng, Jinghua Yan, Hong-Wei Wang, Qihui Wang, Jianxun Qi, George Fu Gao

**Affiliations:** ^a^Chinese Academy of Sciences Key Laboratory of Pathogenic Microbiology and Immunology, Institute of Microbiology, Chinese Academy of Sciences, 100101 Beijing, China;; ^b^University of Chinese Academy of Sciences, 100049 Beijing, China;; ^c^Faculty of Health Sciences, University of Macau, 999078 Macau SAR, China;; ^d^College of Veterinary Medicine, Shanxi Agricultural University, 030801 Jinzhong, China;; ^e^Ministry of Education Key Laboratory of Protein Sciences, Tsinghua-Peking Joint Center for Life Sciences, Beijing Advanced Innovation Center for Structural Biology, Beijing Frontier Research Center of Biological Structures, School of Life Sciences, Tsinghua University, 100084 Beijing, China;; ^f^Chinese Academy of Sciences Key Laboratory of Microbial Physiological and Metabolic Engineering, Institute of Microbiology, Chinese Academy of Sciences, 100101 Beijing, China;; ^g^Laboratory of Protein Engineering and Vaccines, Tianjin Institute of Industrial Biotechnology, Chinese Academy of Sciences, Tianjin 300308, China;; ^h^Institute of Physical Science and Information, Anhui University, 230039 Hefei, China;; ^i^Savaid Medical School, University of Chinese Academy of Sciences, 100049 Beijing, China

**Keywords:** COVID-19, SARS-CoV-2, *Rhinolophus macrotis*, ACE2, RBD

## Abstract

The coronavirus disease 2019 (COVID-19) pandemic caused by severe acute respiratory syndrome coronavirus 2 (SARS-CoV-2) has emerged as a major threat to global health. Although varied SARS-CoV-2–related coronaviruses have been isolated from bats and SARS-CoV-2 may infect bat, the structural basis for SARS-CoV-2 to utilize the human receptor counterpart bat angiotensin-converting enzyme 2 (bACE2) for virus infection remains less understood. Here, we report that the SARS-CoV-2 spike protein receptor binding domain (RBD) could bind to bACE2 from *Rhinolophus macrotis* (bACE2-Rm) with substantially lower affinity compared with that to the human ACE2 (hACE2), and its infectivity to host cells expressing bACE2-Rm was confirmed with pseudotyped SARS-CoV-2 virus and SARS-CoV-2 wild virus. The structure of the SARS-CoV-2 RBD with the bACE2-Rm complex was determined, revealing a binding mode similar to that of hACE2. The analysis of binding details between SARS-CoV-2 RBD and bACE2-Rm revealed that the interacting network involving Y41 and E42 of bACE2-Rm showed substantial differences with that to hACE2. Bats have extensive species diversity and the residues for RBD binding in bACE2 receptor varied substantially among different bat species. Notably, the Y41H mutant, which exists in many bats, attenuates the binding capacity of bACE2-Rm, indicating the central roles of Y41 in the interaction network. These findings would benefit our understanding of the potential infection of SARS-CoV-2 in varied species of bats.

## Significance

It is widely believed that SARS-CoV-2 may infect bats, but direct evidence is still lacking and the molecular basis is less understood. Here, we report that SARS-CoV-2 receptor binding domain (RBD) binds to bACE2-Rm with lower affinity than that to human ACE2 receptor (hACE2). Pseudotyped and wild SARS-CoV-2 could infect host cells expressing bACE2-Rm. The complex structure of SARS-CoV-2 RBD and bACE2-Rm revealed a conserved binding mode similar to that of hACE2. Mutational analysis revealed that the Y41 and E42 of bACE2-Rm, which contains variations in many bats, play central roles in the interaction with SARS-CoV-2 RBD. These findings provide the molecular basis for a better understanding of potential infection of SARS-CoV-2 in bats.

The coronavirus disease 2019 (COVID-19), caused by infection with the novel coronavirus (CoV) severe acute respiratory syndrome coronavirus 2 (SARS-CoV-2), has emerged as a major threat to global health with an increasing number of infected cases globally, and the end to this pandemic is still full of uncertainties (1–3). Seven CoVs have been reported to infect humans: SARS-CoV, Middle East respiratory syndrome coronavirus (MERS-CoV), NL-63, OC43, 229E, HKU1, and the newly emerging SARS-CoV-2. Among the SARS-CoV-2 proteins, the spike (S) protein, which consists of an N-terminal S1 subunit and a C-terminal S2 subunit, is critical for the recognition of host cell receptors and serves as the key determinant of host specificity for CoVs (4). The C-terminal domain of the S1 subunit, also known as the receptor binding domain (RBD), binds to the human angiotensin-converting enzyme 2 (hACE2), the receptor for SARS-CoV and the human coronavirus NL-63 (5–9). Monoclonal antibodies (mAbs) that block the binding between SARS-CoV-2 RBD and ACE2 could efficiently inhibit virus infection in host cells expressing ACE2 (10, 11). Animal studies revealed that a single dose of these neutralizing mAbs showed promising therapeutic efficacy in reducing both viral load and pathological lung damage in hACE2 transgenic mice or rhesus macaques (10, 11). Recently, the structures of the complex between SARS-CoV-2 S protein (or RBD) with hACE2 have been determined, showing similar binding mode to that of SARS-CoV but with enhanced affinity (5–7).

Bats are considered as the reservoir host animals of SARS-CoV-2, and several SARS-CoV-2-related CoVs have been identified from bats (12, 13). The binding assay showed that bat ACE2 (bACE2) from *Rhinolophus macrotis* (bACE2-Rm) bound to SARS-CoV-2 RBD efficiently (14). The entry capacity of SARS-CoV-2 through ACE2 orthologs from 46 bat species was evaluated by virus-host receptor binding and infection assays. The results indicated that although some bACE2 receptors could mediate SARS-CoV-2 entry, there are many bACE2 receptors that do not yet support SARS-CoV-2 entry (15). To date, the genome most closely related to SARS-CoV-2 is RaTG13, which was identified from a *Rhinolophus affinis* sampled from Yunnan Province in 2013 and had a 92.9% amino acid identity in the S gene (13). RmYN02 was also a coronavirus identified from bat with 93.3% nucleotide identity with SARS-CoV-2 at the scale of the complete virus genome (12). Yongyi Shen and colleagues (16) reported a coronavirus isolated from Malayan pangolin and shared 90.7% amino acid identity with SARS-CoV-2 in the S proteins. In addition, a coronavirus that showed 97.4% amino acid identity with SARS-CoV-2 in the RBD region was identified from another batch of Malayan pangolin (17). Bioinformatics analysis indicated that bats are the primary reservoir for the SARS-CoV-2 lineage (18). However, the origin of SARS-CoV-2 is full of uncertainties and whether or not there is an intermediate host is still unknown. Moreover, more than 1,400 species of bats have been identified and extensive species diversity among different species of bats has resulted in varied susceptibility for CoVs. Research into the mechanisms of viral entry and virus–host interaction would not only benefit our understanding of this virus but is also important for the design of antivirals and vaccines. However, the structure of bACE2 has not been determined, and the molecular basis of the binding between SARS-CoV-2 S protein and bACE2 has not been well studied.

Here, we report that SARS-CoV-2 RBD can bind to bACE2-Rm with substantially lower affinity than that to hACE2, and infection of host cells carrying bACE2-Rm was also investigated with pseudotyped or wild SARS-CoV-2. Interaction mechanisms between SARS-CoV-2 RBD and bACE2-Rm were elucidated by determining the structure of the SARS-CoV-2 RBD and bACE2-Rm complex. The results of this study would broaden our understanding of the receptor binding mechanisms for SARS-CoV-2.

## Results

### Binding of SARS-CoV-2 RBD to bACE2-Rm and Infectivity of Pseudotyped SARS-CoV-2.

**Fig. 1.**
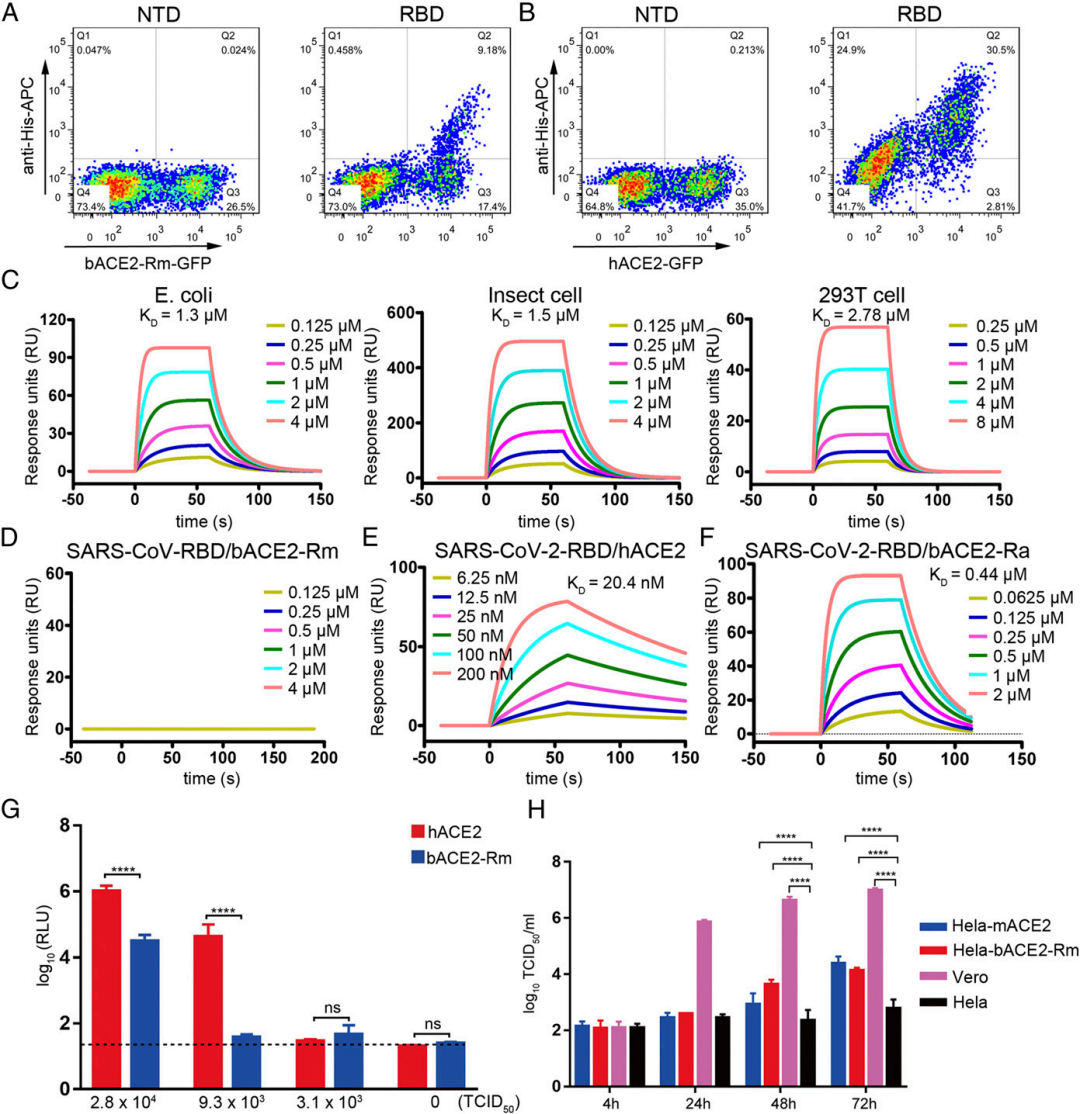
Binding between SARS-CoV-2 RBD and bACE2-Rm, and the infectivity of pseudotyped SARS-CoV-2 in BHK21 cells expressing bACE2-Rm. (*A* and *B*) Flow cytometric assay of SARS-CoV-2 RBD binding the bACE2-Rm (*A*) or hACE2 (*B*) expressed on the surface of HEK293T cells. Binding with NTD was included as negative control. (*C*) SPR characterization of the binding between the SARS-CoV-2 RBD and the bACE2-Rm proteins obtained from the indicated cells. (*D*) Binding between SARS-CoV RBD and bACE2-Rm proteins obtained from insect cells. (*E*) Binding between SARS-CoV-2 RBD to hACE2. (*F*) Binding between SARS-CoV-2 RBD to bACE2-Ra. (*G*) Infectivity of the pseudotyped SARS-CoV-2 infecting BHK21 cells expressing the bACE2-Rm or hACE2. Different titers of pseudotyped SARS-CoV-2 were used to infect BHK21 cells. Error bars represent the SEM from four independent assays. (*H*) Viral growth kinetics with SARS-CoV-2 to infect HeLa cells stably expressing bACE2-Rm (HeLa-bACE2-Rm). HeLa cells stably expressing monkey ACE2 (HeLa-mACE2) and Vero cells with endogenous monkey ACE2 expression (Vero) were used as positive control, while HeLa cells without ACE2 expression (HeLa) were used as negative control. ****P < 0.0001, student’ s *t* test. ns, no significant differences.

We first tried to evaluate the binding capacity of bACE2-Rm, a representative bat species widely distributed in Southeast Asia, to SARS-CoV-2 RBD through a flow cytometry-based binding assay. The results showed that the SARS-CoV-2 RBD could bind to bACE2-Rm–expressing HEK293T cells with a substantially lower positive rate than that of hACE2, with 34.5% bACE2-Rm–expressing cells that stained positive versus 91.6% hACE2-expressing cells that stained positive (Fig. 1 *A* and *B*). bACE2-Rm contains six potential N-linked glycosylation sites (N53, N90, N329, N546, N660, and N690), and the structure of hACE2 has been revealed to be highly glycosylated (19, 20). A previous study has reported that the glycosylation of proteins varies in different expression systems (21); for example, proteins are nonglycosylated in *Escherichia coli*-based expression platforms (22). The glycosylation compositions of proteins are substantially different when expressed in HEK293T cells and insect cells (23, 24). Therefore, using surface plasmon resonance (SPR) analysis, we further tested the binding of the SARS-CoV-2 RBD with bACE2-Rm proteins expressed in HEK293T cells, insect cells, or refolded from inclusion bodies obtained from *E. coli*, which all carry different levels of glycosylation modifications (23). The results showed that the SARS-CoV-2 RBD could bind to the bACE2-Rm expressed in HEK293T cells with an affinity (*K*_D_) of 2.78 μM, while no substantial difference was observed compared with that of the bACE2-Rm proteins obtained from insect cells (1.5 μM) or *E. coli* (1.3 μM) (Fig. 1*C* and *SI Appendix*, Table S1). This result indicates that the binding of SARS-CoV-2 RBD with bACE2-Rm is independent on bACE2-Rm glycosylation modifications. As controls, the binding affinity between SARS-CoV RBD and bACE2-Rm (no binding), and the binding between SARS-CoV-2 RBD and hACE2 (*K*_D_ = 20.4 nM) (Fig. 1 *D* and *E*), are both consistent with previous studies (5–7, 25). The binding affinity of SARS-CoV-2 RBD and bACE2 from *R. affinis* (bACE2-Ra; *K*_D_ = 0.44 μM), from which the most similar RaTG13 was identified, was found to be substantially higher than that of the bACE2-Rm (Fig. 1*F*).

To investigate whether SARS-CoV-2 infects host cells via binding to bACE2-Rm, a pseudotyped virus infection system carrying the SARS-CoV-2 S protein was employed to transduce BHK21 cells for transient expression of either bACE2-Rm or hACE2 (26). The results showed that pseudotyped SARS-CoV-2 could infect BHK21 cells expressing bACE2-Rm with substantially lower infectivity to hACE2 (Fig. 1*G*). Moreover, the growth kinetics of wild SARS-CoV-2 were further investigated using HeLa cells stably expressing bACE2-Rm, with HeLa cells stably expressing monkey ACE2 (which is most similar to hACE2, with its residues contacting SARS-CoV-2 RBD highly conserved with hACE2), and Vero cells with endogenous monkey ACE2 expression as a positive control. Infection of the SARS-CoV-2 to HeLa cells expressing monkey ACE2 or bACE2-Rm could be detected after 48 h of incubation, while no SARS-CoV-2 could be detected in HeLa cells without ACE2 receptor expression (Fig. 1*H*). Therefore, SARS-CoV-2 could bind to bACE2-Rm with lower binding affinity than that of hACE2 and infects host cells via binding to bACE2-Rm.

### Conserved Structure of the bACE2-Rm Homodimer.

**Fig. 2.**
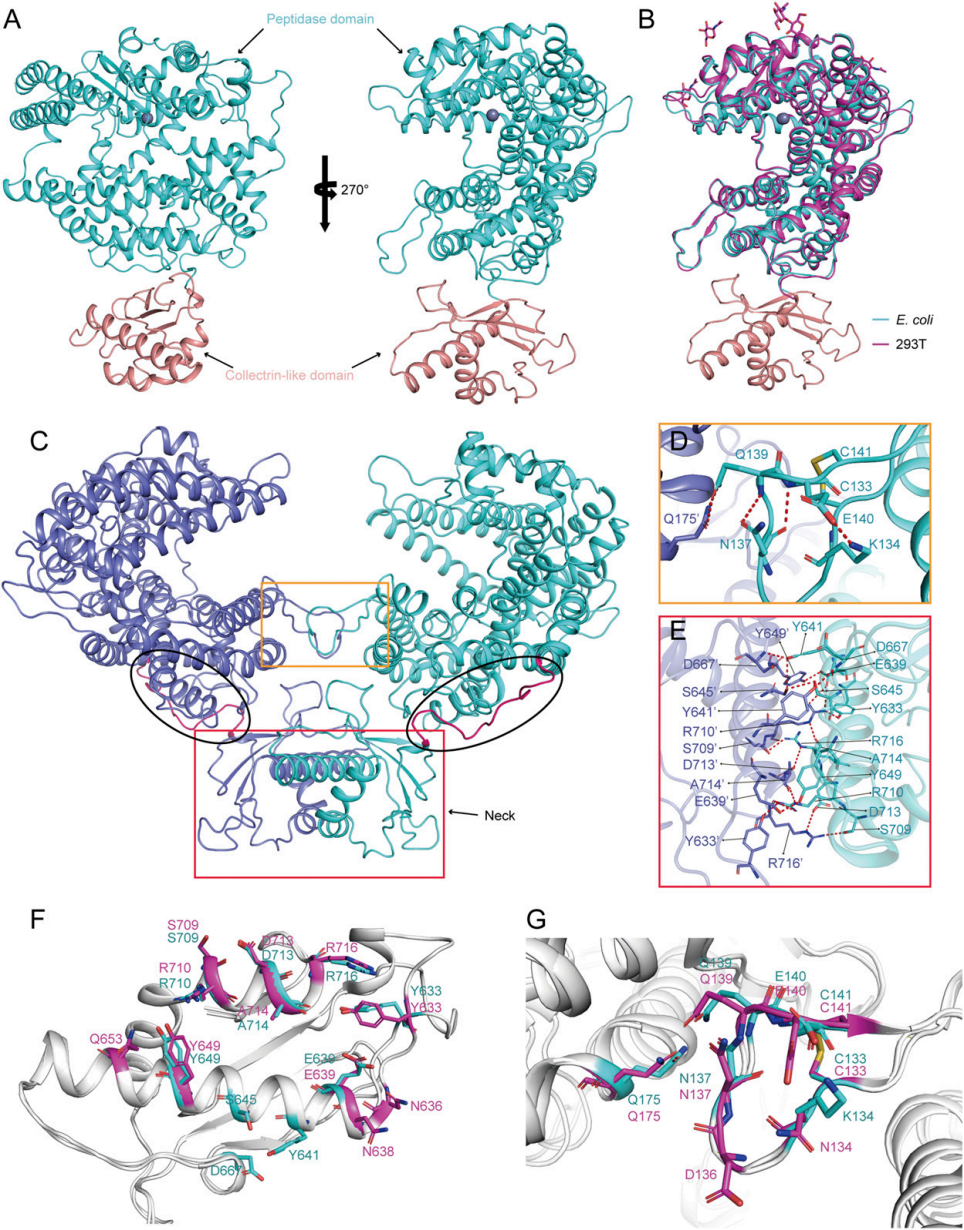
The structure of the bACE2-Rm and the molecular basis of the bACE2-Rm dimer. (*A*) Overall structure of the bACE2-Rm with an N-terminal distal peptidase domain and a CLD, colored in teal and wheat, respectively. (*B*) Superimposition of the bACE2-Rm (expressed from *E. coli* cells) structure obtained from the complex structure and the cryo-EM bACE2-Rm (expressed from HEK293T cells) structure. (*C*) Representation of the bACE2-Rm dimer, highlighting the regions forming the dimeric interface in the CLD and the loops in PD with rectangles. (*D* and *E*) Detailed interaction within the minor dimeric interface located in the PD (*D*) and the major dimeric interface within the CLD (*E*). Residues involved in the hydrogen bond interaction are shown as sticks and are labeled. Hydrogen bonds are shown as dashed black lines. (*F* and *G*) The conservation analysis of the residues involved in the formation of the ACE2 homodimer between humans (pink) and bats (cyan).

Complex proteins of SARS-CoV-2 RBD and bACE2-Rm were prepared, with the bACE2-Rm proteins obtained from**in vitro refolding of the inclusion bodies expressed in *E. coli* cells and set up for crystal screening (*SI Appendix*, Fig. S1). The SARS-CoV-2 RBD/bACE2-Rm complex structure was determined at a resolution of 3.2 Å (*SI Appendix*, Table S2). Overall, the structure of the bACE2-Rm extracellular domain is composed of an N-terminal peptidase domain (PD) and a C-terminal collectrin-like domain (CLD), which is similar to hACE2 (Fig. 2*A*). Superimposition of the PD of bACE2-Rm and hACE2 yields a root mean square deviation (RMSD) of 0.711 Å for 559 equivalent Cα atoms, indicating the highly conserved conformation of bACE2-Rm. The cryoelectron microscopy (cryo-EM) structure of bACE2-Rm, which was expressed in HEK293T cells, was also determined, which showed no substantial difference with bACE2-Rm from the SARS-CoV-2 RBD/bACE2-Rm complex crystal structure (Fig. 2*B* and *SI Appendix*, Table S3). The homodimer of bACE2-Rm was observed through a simple symmetric operation (Fig. 2*C*). Dimerization is mainly mediated by the CLD, while the PD contributes a minor interface. An extensive hydrogen bond network was formed between the two CLDs involving the amino acids from the helices, while the minor interface was mainly mediated by hydrogen bond interaction between Q139 and Q175′ of the PD (Fig. 2 *D* and *E*). A conservation analysis of the residues involved in the formation of the ACE2 homodimer revealed that most residues are conserved between humans and bats, indicating that the ACE2 dimer is conserved during evolution (Fig. 1 *F* and *G*).

### Interaction between SARS-CoV-2 RBD and bACE2-Rm.

**Fig. 3.**
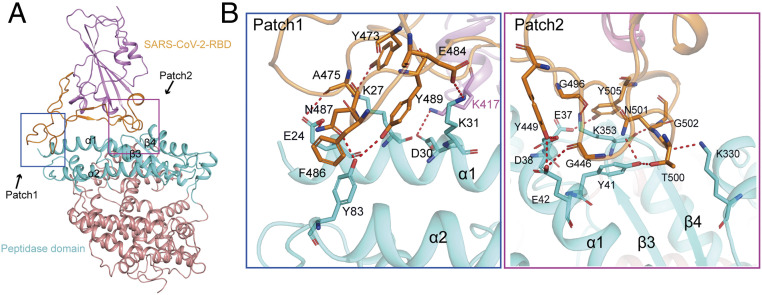
Structural basis of the binding between the SARS-CoV-2 RBD and bACE2-Rm. (*A*) Overall structure of SARS-CoV-2 RBD and bACE2-Rm. (*B*) Details of the binding between SARS-CoV-2 RBD and bACE2-Rm. Binding between the RBD external domain and bACE2-Rm is mainly composed of two patches of interactions, Patch 1 and Patch 2. Patch 1 interaction mainly involves the interaction with α1 and α2 (*Left*), while Patch 2 interactions are mainly located on a conformational surface comprising residues from α1, β-hairpin, and a small distal helix (*Right*). Residues in the hydrogen bonds are shown as sticks and are labeled. Hydrogen bonds are shown as dashed black lines.

The complex structure of SARS-CoV-2 RBD with bACE2-Rm showed that the RBD binds to bACE2-Rm with its external subdomain composed of large polypeptide loops with two small β-strands located distally, cradling the core subdomain (Fig. 3*A*). The binding of the SARS-CoV-2 RBD to bACE2-Rm is distributed into two patches, with Patch 1 located on the N-terminal α1 and α2, and Patch 2 located on a conformational surface consisting of residues from α1, a β-hairpin constituted by β3 and β4, and a small distal helix (4). Overall, the complex buried a surface area comparable to that of hACE2, 1773 Å^2^ vs. 1761 Å^2^, respectively (5). In the Patch 1 interaction network, residues from α1 (E24, K27, D30, and K31) and α2 (Y83) of bACE2-Rm formed a hydrogen bond network with amino acids (Y473, A475, E484, N487, and Y489) from the long extending loop connecting the two short β-strands of the external domain of the SARS-CoV-2 RBD. In Patch 2, amino acids (Y449, G496, N501, G502, and Y505) from the extending loop of the external domain of the SARS-CoV-2 RBD formed multiple hydrogen bonds with residues from α1 (E37, D38, Y41, and E42), K353 from the β-hairpin, and K330 from the small external helix (Fig. 3*B* and *SI Appendix*, Table S4).

### Molecular Basis of the Cross-Species Interaction between SARS-CoV-2 RBD and bACE2-Rm.

**Fig. 4.**
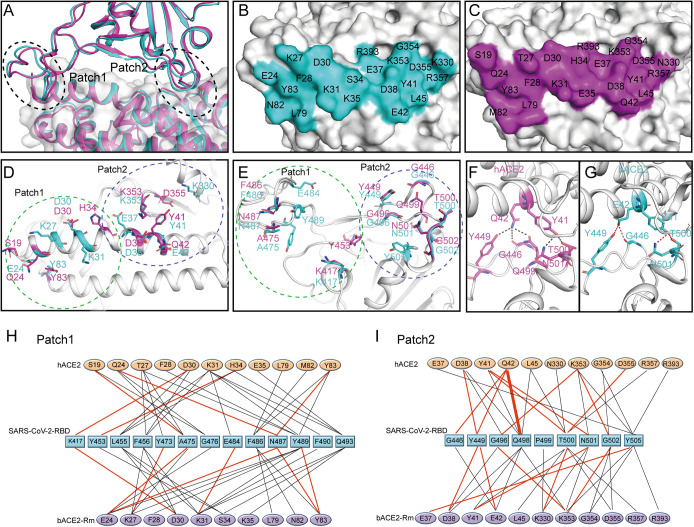
Comparison of the binding between bACE2-Rm and hACE2, and identification of determinants between RBD and the bACE2-Rm. (*A*) An overall view of the interface between RBD and ACE2 by superimposing the two SARS-COV-2 RBD structures with bACE2-Rm or hACE2. The complex of SARS-CoV-2 RBD with either bACE2-Rm or hACE2 is colored in teal or purple, respectively. (*B* and *C*) The buried surfaces on the bACE2-Rm (*B*) or hACE2 (*C*) by the SARS-CoV-2 RBD, with contacting residues labeled accordingly. (*D*) Amino acids in bACE2-Rm (teal) or hACE2 (purple) in the hydrogen bonds with SARS-CoV-2 RBD are represented as sticks. (*E*) Residues in the SARS-CoV-2 RBD that contact with bACE2-Rm (teal) or hACE2 (purple) with hydrogen bonds. (*F* and *G*) The interaction network of the Y41 and Q42 in hACE2 (*F*), and the Y41 and Q42 of the bACE2-Rm with the SARS-CoV-2 RBD (*G*). (*H* and *I*) Residues involved in the interaction of the SARS-CoV-2 RBD with bACE2-Rm or hACE2 are listed and connected by solid lines. Black lines indicate van der Waals contacts, while red lines represent H-bonds or salt bridges.

The previously reported SARS-CoV-2-RBD/hACE2 complex structures enabled us to compare interaction details. Superimposition of the α1 and α2 of bACE2-Rm and hACE2 from the two complexes, which are the primary regions responsible for the interaction with SARS-CoV-2 RBD, yielded an RMSD of 0.753 over 739 Cα atoms, indicating that the overall topology of the two complexes resemble each other (Fig. 4*A*). The binding areas of the SARS-CoV-2 RBD on bACE2-Rm and hACE2 are also similar to each other (Fig. 4 *B* and *C*). The residues contacting SARS-CoV-2 RBD in Patch 1 are substantial differences between bACE2-Rm and hACE2 (Fig. 4 *D* and *E*). On the other hand, the interacting network in Patch 2 showed highly conserved.

Within the interface with the SARS-CoV-2 RBD, Y41 and E42 of bACE2-Rm play a central role, while hACE2 carries Y41 and Q42 in the corresponding region (27). Of note, these two residues also account for the major differences in the interaction network with SARS-CoV-2 RBD between bACE2-Rm and hACE2 (Fig. 4 *F*–*I*). *R. macrotis*, a species of bat belonging to the *Rhinolophidae* family, is widely distributed in China, India, and Pakistan, as well as in Southeast Asian countries (*SI Appendix*, Fig. S2) (28). Based on the fact that bats have extensive species diversity, the binding capacity of bACE2-Rm to SARS-CoV-2 RBD may vary among species. Sequence alignment of the key residues in bACE2-Rm involved in interaction with SARS-CoV-2 RBD from 32 bat species was carried out (29). The results showed that H41 and Q42 variants were present in many bats (*SI Appendix*, Table S5). Therefore, we speculate that binding to Y41 and E42 of bACE2-Rm may be the determinant residues within the interaction.

**Fig. 5.**
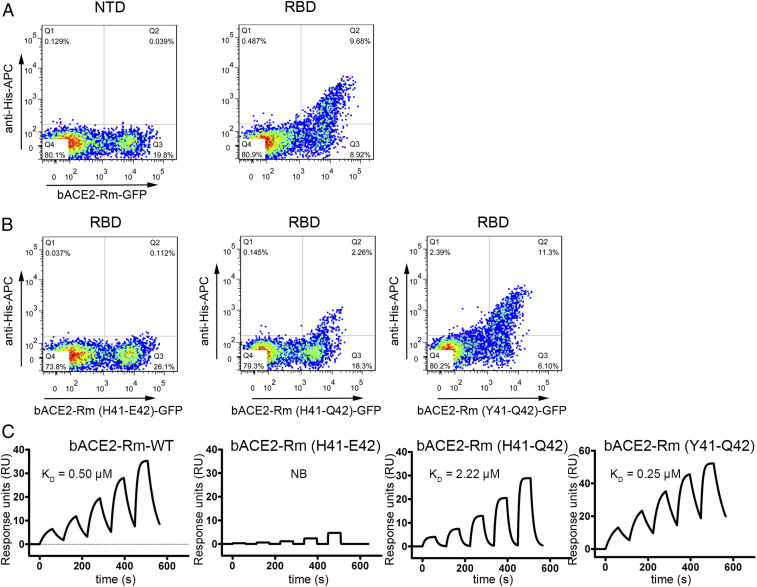
Mutational analysis of the key residues in bACE2-Rm involved in the interaction with SARS-CoV-2. (*A* and *B*) Flow cytometry-based assay characterizing the binding of the SARS-CoV-2 RBD with wild-type bACE2-Rm, bACE2-Rm (H41-E42), bACE2-Rm (H41-Q42), or bACE2-Rm (Y41-Q42) mutants expressed on the surface of HEK293T cells as indicated. (*C*) SPR analysis of the binding of the SARS-CoV-2 RBD with bACE2-Rm-WT, bACE2-Rm (H41-E42), bACE2-Rm (H41-Q42), or bACE2-Rm (Y41-Q42) mutant proteins.

Mutational analysis was further conducted by transiently expressing bACE2-Rm carrying the Y41H and/or E42Q mutations in HEK293T cells. The results showed that the binding capacity of SARS-CoV-2 RBD to bACE2-Rm is attenuated with the introduction of the Y41H mutation (Fig. 5*B* and *SI Appendix*, Fig. S3). SPR analysis showed that no binding could be observed with the bACE2-Rm (H41-E42) mutant (Fig. 5*C*). In contrast, introducing Q42 to the bACE2-Rm (H41-E42) mutant, which results in bACE2-Rm (H41-Q42), rescued its binding capacity to SARS-CoV-2 RBD (*K*_D_ = 2.22 μM), whereas mutation of E42Q in the wild-type bACE2-Rm resulted in a slightly higher binding affinity to SARS-CoV-2 RBD (*K*_D_ = 0.25 μM) (Fig. 5 *B* and *C*). Staining of SARS-CoV-2 RBD with HEK293T cells expressing bACE2-Rm from *Rhinolophus sinicus*, which carries H41-Q42 and is widely distributed in southern China, Nepal, northern India, and Vietnam as *R. macrotis*, showed that no substantial binding could be observed (*SI Appendix*, Fig. S4). Therefore, the interaction network with Y41 is crucial for the binding between SARS-CoV-2 RBD and bACE2-Rm, which may also determine the host range of SARS-CoV-2.

## Discussion

In this study, we demonstrated that SARS-CoV-2 RBD could bind to bACE2-Rm and infect host cells expressing bACE2-Rm in the pseudotyped or wild SARS-CoV-2 infection system. The binding affinity between SARS-CoV-2 RBD and bACE2-Rm is substantially lower than that between SARS-CoV-2 RBD and hACE2, which is also supported by the lower infection efficacy with pseudotyped SARS-CoV-2. The capacity of virus infection was further confirmed with SARS-CoV-2 wild virus to infect HeLa cells stably expressing bACE2-Rm. Guo et al. (30) recently investigated the binding and infection of a series of SARS-related CoVs isolated from *R. sinicus* with ACE2 variants carrying polymorphic sites involved in the interaction with S protein, suggesting a long-term and ongoing coevolutionary dynamics between the S protein and ACE2 receptor. Additionally, two groups isolated SARS-CoV-2–related CoVs from Malayan pangolins, with one CoV showing a 90.7% identity of S genes and a virtually identical RBD with SARS-CoV-2 (16, 31). Therefore, it is reasonable to speculate that the SARS-CoV-2–like CoVs may widely exist in wild animals.

The structure of the SARS-CoV-2 RBD and bACE2-Rm complex is determined, which is overall conserved with the structure of the SARS-CoV-2 RBD and hACE2 complex. The structure of bACE2-Rm is highly conserved with that of hACE2, providing the structural basis for cross-species interaction of CoVs originating from bats. Comparative analysis of the structures of SARS-CoV-2 RBD and bACE2-Rm complex with SARS-CoV-2 RBD and hACE2 complex revealed that the contacting residues of SARS-CoV-2 RBD involved in the interaction with bACE2-Rm is conserved with that of hACE2. Major differences exist in the Patch 2 interaction network, which may also be responsible for the lower binding affinity with bACE2-Rm. Bats are considered to be the reservoir hosts for many viruses, including CoVs. The extensive species diversity of the host receptors among different species of bats may have made bats a complicated reservoir for viral evolution. Structural analysis revealed that the Y41 and E42 of bACE2-Rm, which are involved in the Patch 2 interaction network, play critical roles in the formation of the hydrogen-bond interaction network with SARS-CoV-2 RBD. Sequence analysis of bACE2-Rm from various bats revealed that this region is highly polymorphic, with H41-E42, H41-Q42, and Y41-Q42 variants existing in different bats. Both flow cytometry analysis and SPR assay support that bACE2-Rm receptors carrying these variants have shown varied binding capacity to SARS-CoV-2 RBD. Although the geographical distribution of different species of bats extensively overlapped with each other, the capacity of different bats to carry SARS-CoV-2 may vary substantially from each other. This also raises the possibility that the extensively diversified bACE2 may have profound effects on the evolutionary adaption of SARS-CoV-2 RBD to other potential intermediate host receptors for cross-species transmission.

A growing body of research has identified that the binding capacity of ACE2 orthologs from different bat species to RBD of SARS-CoV or SARS-CoV-2 varied substantially, indicating the extensively diversified susceptibility of SARS-CoV and SARS-CoV-2 in different bat species (18, 25, 32, 33). Guo et al. (30) reported that ACE2 genes showed high polymorphism among the *R. sinicus* populations, and *R. sinicus* ACE2 variants possessed different susceptibility to SARS-related-CoV infection. In the present study, together with the results from our previous work, our data showed that *R. sinicus* ACE2 could not bind to SARS-CoV-2 RBD (34). Zhao and colleagues (15) also reported *R. sinicus* ACE2 could not bind to SARS-CoV-2 RBD, although another report showed positive binding between *R. sinicus* ACE2 and SARS-CoV-2 RBD (13). These contradictory results may be due to the high polymorphism of *R. sinicus* ACE2 receptors, which should be addressed in the future.

In summary, we found that SARS-CoV-2 could bind to bACE2-Rm and infect host cells expressing bACE2-Rm with pseudotyped and wild SARS-CoV-2. The structure of the SARS-CoV-2 RBD and bACE2-Rm complex was determined, revealing a binding mode similar to that of hACE2. Y41 and E42 of bACE2-Rm play critical roles in the interaction network with SARS-CoV-2 RBD; bACE2-Rm mutant proteins, which carry polymorphism residues at these two sites presented in different species of bats, have shown varied binding capacity with SARS-CoV-2 RBD. These findings provide evidence that SARS-CoV-2 may infect bats, and that the extensive species diversity of bats may have profound effects on SARS-CoV-2 evolution. Overall, our results shed light for future surveillance of bat-originated CoVs.

## Methods

### Gene Cloning.

Genes encoding bACE2-Rm from *R. macrotis* (GenBank: ADN93471.1) expressed in different expression systems were constructed. For expression in *E. coli*, residues 19 to 741 of bACE2-Rm were cloned into the pET21a vector. For the Bac-to-Bac baculovirus expression system, bACE2-Rm (residues 19 to 615), SARS-CoV-2-NTD (N-terminal domain of spike protein) (residues 1 to 286, GISAID: EPI_ISL_402119), SARS-RBD (residues 306 to 527, GenBank: NC_004718), and SARS-CoV-2-RBD (residues 319 to 541, GISAID: EPI_ISL_402119) were constructed individually and purified as previously reported (5). Briefly, genes were cloned into pFastbac1 vector (Invitrogen) with an N-terminal gp67 signal peptide for secretion and a C-terminal hexa-His tag for purification. For HEK293T cell expression, residues 19 to 615 of bACE2-Rm fused to the mouse Fc tag (bACE2-Rm-mFc) were cloned into the pCAGGS vector.

### Protein Expression and Purification.

bACE2-Rm-*E. coli* expression proteins were expressed as inclusion bodies. The inclusion bodies of bACE2-Rm were purified and then refolded as previously described (35–38). After refolding, proteins were concentrated and exchanged into a buffer (20 mM Tris⋅HCl, pH 8.0, 150 mM NaCl) and purified using a Superdex 200 Increase 10/300 GL column (GE Healthcare). For the Bac-to-Bac baculovirus expression system, recombinant baculovirus-containing target genes were used to infect Hi5 cells to produce soluble proteins. The protein was purified using a His-Trap HP column (GE Healthcare) after the supernatant was collected and filtered through a 0.22-μm filter membrane and then further purified using a Superdex 200 Increase 10/300 GL column (GE Healthcare). For HEK293T cell expression, pCAGGS plasmids containing bACE2-Rm-mFc were transiently transfected into cells. After 4-d expression, supernatants were collected, centrifuged, and mixed with the same volume of binding buffer containing 20 mM Na_3_PO_4_ (pH 7.0). The mixtures were then filtered through 0.22-mm filters and passed through a HiTrap Protein A FF (GE Healthcare) affinity chromatography column. The bound proteins were eluted with 0.1 M glycine-HCl (pH 3.0) and collected into tubes containing 200 mL of 1 M Tris⋅HCl (pH 9.0). The proteins were then exchanged into protein buffer (20 mM Tris⋅HCl, pH 8.0, 150 mM NaCl) and further purified by Superdex 200 Increase 10/300 GL column (GE Healthcare).

### SPR Analysis.

All SPR measurements were performed using a BIAcore 8000 system (GE Healthcare) with CM5 chips (GE Healthcare). For all measurements, HBST (20 mM Hepes, 150 mM NaCl, pH 7.4) was used as the running buffer. All of the tested proteins were transferred to HBST. To detect SARS-CoV-2-RBD binding to hACE2 and bACE2-Rm proteins obtained from different expression systems, bACE2-Rm-*E. coli* (1,585 units), bACE2-Rm-insect (6,943 units), bACE2-Rm-HEK293T cells (1,068 units), and hACE2-insect (5,416 units) were immobilized on the CM 5 chip. SARS-CoV-2-RBD was serially diluted to concentrations ranging from 0.125 μM to 4 μM and were then flowed over bACE2-Rm-*E. coli* and bACE2-Rm-insect channels, respectively. The concentrations ranging from 0.25 μM to 8 μM of SARS-CoV-2-RBD were flowed over the bACE2-Rm-HEK293T cell channel. The concentrations ranging from 31.25 nM to 1 μM of SARS-CoV-RBD were flowed over the bat ACE2-insect channel (1614.2 units). The concentrations ranging from 6.25 nM to 200 nM of SARS-CoV-2-RBD were flowed over the human ACE2 channel. After each cycle, the sensor surface was regenerated with HBST buffer. The binding affinity of bACE2-Rm carrying the Y41H and/or E42Q mutations to SARS-CoV-2-RBD was also evaluated by SPR. The mouse Fc (mFc)-tagged ACE2s (bACE2-Rm (H41-E42), bACE2-Rm (H41-Q42), and bACE2-Rm (Y41-Q42)) were captured using a chip preimmobilized with anti-mouse IgG antibodies, and then the serially diluted SARS-CoV-2-RBD proteins were flowed through the chip. The binding kinetics were analyzed using the software of Biacore Insight evaluation software (GE Healthcare) using a 1:1 Langmuir binding model.

Concentrated supernatants containing bACE2-Rm-mFc and bACE2-Rm-Y41H-mFc were individually captured by the antibody immobilized on the CM5 chip. The concentrations of SARS-CoV-2-RBD diluted to 250, 500, 1,000, 2,000, and 4,000 nM were then flowed through the chip and the real-time response was recorded. The binding kinetics were analyzed using the Biacore Insight evaluation software (GE Healthcare) using a 1:1 Langmuir binding model.

### Flow Cytometry.

For the flow cytometry assay, bACE2-Rm, bACE2-Rm-Y41H, and hACE2 fused with eGFP were transfected into HEK293T cells. Cells were harvested after 24 h and then suspended in PBS (with 0.5% FBS) and incubated with the test proteins (SARS-CoV-2-RBD and SARS-CoV-2-NTD) with a histidine tag at 37 °C for 30 min. Cells were washed three times with PBS and further incubated with anti-His/APC antibodies (1:500, Miltenyi Biotec) at 37 °C for 30 min. After washing, the cells were analyzed using a BD FACSCalibur instrument. The figures were generated using FlowJo 7.6.

### Pseudovirus Transduction.

Pseudotyped SARS-CoV-2 particles were provided by the Academy of Military Medical Sciences. For pseudovirus transduction, pEGFP-N1-bACE2-Rm and pEGFP-N1-hACE2 plasmids fused to eGFP were transferred to BHK21 cells. After 24 h, eGFP^+^ cells were sorted by flow cytometry. Then, eGFP^+^ cells (4 × 10^4^ cells per well) were seeded in 96-well plates for 24 h. The TCID_50_ of pseudovirus particles was serially diluted three folds ranging from 2.8 × 10^4^ to 3.1 × 10^4^. Then, the supernatant containing pseudovirus particles was added to the BHK21 cells after PBS washing. Cells were lysed in the Luciferase Assay System reagent (Promega) at 24 h postinfection. Luciferase activity was detected using a GloMax 96 Microplate luminometer (Promega).

### Crystallization, Data Collection, and Structure Determination.

To obtain high-resolution crystals, the sitting-drop method was used. In detail, purified bACE2-Rm-*E. coli*/SARS-CoV-2-RBD proteins were concentrated to 6 mg/mL. Then, 0.8 μL protein was mixed with 0.8-μL reservoir solution. The resulting solution was sealed and equilibrated against 100 μL of reservoir solution at 18 °C. Crystals of bACE2-Rm-*E. coli*/SARS-CoV-2-RBD were grown in 0.1 M succinic acid pH 7.0, 0.1 M BICINE pH 8.5, and 30% (vol/vol) polyethylene glycol monomethyl ether 550 approximately 1 mo later.

Diffraction data were obtained from the Shanghai Synchrotron Radiation Facility BL19U. For data collection, the crystals were picked up from the groove using the mini loop and then soaked in a reservoir solution supplemented with 20% (vol/vol) glycerol for a few seconds. Then, it was picked up and soaked in liquid nitrogen to freeze. The dataset was processed using HKL2000 software (39). Structure of the SARS-CoV-2-RBD/bat ACE2-*E. coli* complex was determined by the molecular replacement method using Phaser (40), with previously reported complex structure SARS-CoV-2-RBD complex with hACE2 (PDB ID code 6LZG). The atomic models were completed with Coot (41) and refined with phenix.refine in Phenix (40), and the stereochemical qualities of the final models were assessed with MolProbity (42). Data collection, processing, and refinement statistics are summarized in *SI Appendix*, Table S2. All structural figures were generated using Pymol software (https://pymol.org/2/).

### Cryo-EM Sample Preparation, Data Collection, Image Processing, and Model Fitting.

The bat-ACE2-Rm/SARS-CoV-2-RBD complex protein was diluted to 0.2 mg/mL. Then, the complex proteins were placed on a glow-discharged home-made graphene grid (Quantifiol Au 1.2/1.3, 300 mesh), allowed to stand for 10 s, blotted for 0.5 s with filter paper, and the grid was plunged into liquid ethane using FEI Vitrobot Mark IV. The cryospecimens were loaded on a 300 kV Titan Krios transmission electron microscope equipped with a GIF-Quantum energy filter and a Gatan K3 direct electron detector for data collection. Images of the samples were exposed to 1.68 s at a normal magnification of 130 k and an electron dose rate of ∼12.9 e^−^ pixel^−1^ s^−1^ using the counting mode, which resulted in a total dose of ∼50 e^−^ Å^−2^ that were fractionated into 32 movie frames. The final defocus range of the datasets was approximately −1.8 to −2.2 μm. The raw dose-fractionated image stacks were 3× Fourier binned, aligned, dose-weighted, and summed using MotionCor2 (43). The initial contrast transfer function (CTF) parameters were estimated using CTFFIND4 (44). Then, we manually selected 2,015 good micrographs from 2,537 raw micrographs based on the Thon ring. All subsequent image processing and reconstruction processes were performed using Relion 3.1 (45). Briefly, we manually picked a set of ∼5,000 particles, which were subjected to two-dimensional (2D) classification to generate templates for reference-based particle picking. A total of 1,272,215 automatically picked particles were extracted with a box size of 160 pixels and rescaled to 80 pixels in Relion 3.1 for the following 2D and three-dimensional (3D) classification. One round of reference-free 2D classification was performed to remove the heterogeneous particles, which yielded poor 2D class average images. A clean dataset with 837,848 particles from good 2D classes was selected and subjected to two further round 3D classifications. After the second round of 3D classification, the predominant class showed the best structural features and the highest accuracy of particle alignment, which contained a subset of 62,289 best particles. Coordinates for these particles were exported to extract the full-size images for final reconstruction. The resulting density map at a resolution of 3.2 Å was determined by the Fourier shell correlation with a cutoff value of 0.143.

From the density map, we could only find bACE2-Rm but not SARS-CoV-2-RBD. For the bACE2-Rm model, the atomic model of hACE2 in the ACE2/SARS-CoV-2-RBD complex (PBD ID code 6LZG) was fit into the electron density map using Chimera (34). The initial structure model was refined against the cryo-EM density map in real space using PHENIX (40) with secondary structure restraints. Automatic real-space and reciprocal-space refinements were performed using COOT (46), and the stereochemical quality of the final model was assessed by MolProbity (47).

### SARS-CoV-2 Wild Virus Infection Assay.

HeLa-monkey-ACE2 cells, HeLa-bACE2-Rm cells, HeLa cells, and Vero cells were inoculated with SARS-CoV-2 at a multiplicity of infection of 0.01, and incubated for 1 h at 37 °C. The virus inoculum was then replaced with fresh Dulbecco’s Modified Eagle’s Medium supplemented with 2% FBS. Culture supernatant was harvested at 4, 24, 48, and 72 h, and viral titer was tested by using quantitative RT-PCR (forward primer: CCC​TGT​GGG​TTT​TAC​ACT​TAA, reverse primer: ACG​ATT​GTG​CAT​CAG​CTG​A, fluorescent probe [P]: 5 ′- the FAM - CCG​TCT​GCG​GTA​TGT​GGA​AAG​GTT​ATG​G- BHQ1-3′). Growth curves are presented as the average value of three independent experiments. Statistical significance was determined by a two-sided unpaired Student's *t* test without adjustments for multiple comparisons.

## Supplementary Material

Supplementary File

## Data Availability

The atomic coordinates for the crystal structure of the SARS-CoV-2 RBD and bACE2-Rm complex and cryo-EM maps of bACE2-Rm have been deposited in the Protein Data Bank (www.rcsb.org) (PDB ID codes 7C8J and 7C8K, respectively).
