# Supplementary material for: Cross-species recognition of SARS-CoV-2 to bat ACE2
Source: Proc Natl Acad Sci U S A. 2020 Dec 28;118(1):e2020216118. doi: 10.1073/pnas.2020216118 (PMC7817217; doi:10.1073/pnas.2020216118)
Supplement: Supplementary File [file pnas.2020216118.sapp.pdf]

**SI Appendix Figures and Tables:**

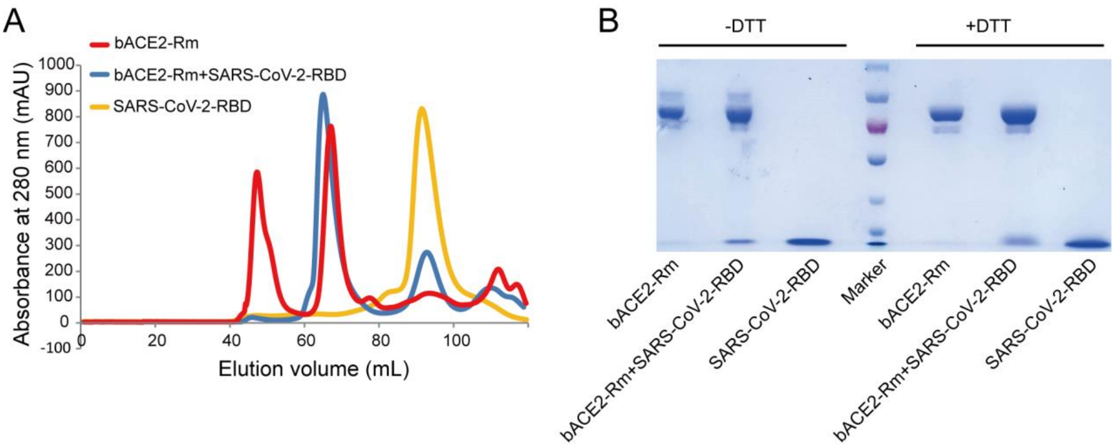


**SI Appendix, Fig. S1. Gel filtration profile of SARS-COV-2-RBD/bACE2-Rm complex protein.** (A) Gel filtration profiles of bACE2-Rm (red), SARS-COV-2-RBD (yellow) and the bACE2-Rm and SARS-COV-2-RBD complex (blue) were analyzed by size-exclusion chromatography as indicated. (B) The separation profiles of each pooled samples on SDS-PAGE are shown in reducing (+DTT) or non-reducing (-DTT) conditions.

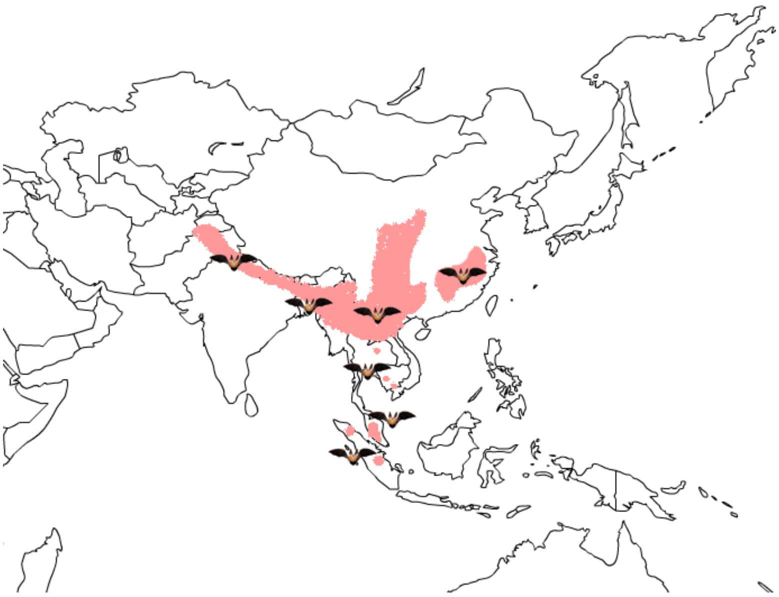

**SI Appendix, Fig. S2. The distribution of *Rhinolophus macrotis*.** *Rhinolophus macrotis*

mainly distribute in Southeast Asia, such as southern China, Bangladesh, India, Indonesia (Sumatra), Lao People's Democratic Republic, Malaysia (Peninsular Malaysia), Myanmar, Nepal, Pakistan, Thailand, Vietnam, Philippines. This figure is generated based on the findings from Vuong Tan Tu, et al. 2017 J Zool Syst Evol Res. (55) 177-198. doi.org/10.1111/jzs.12169

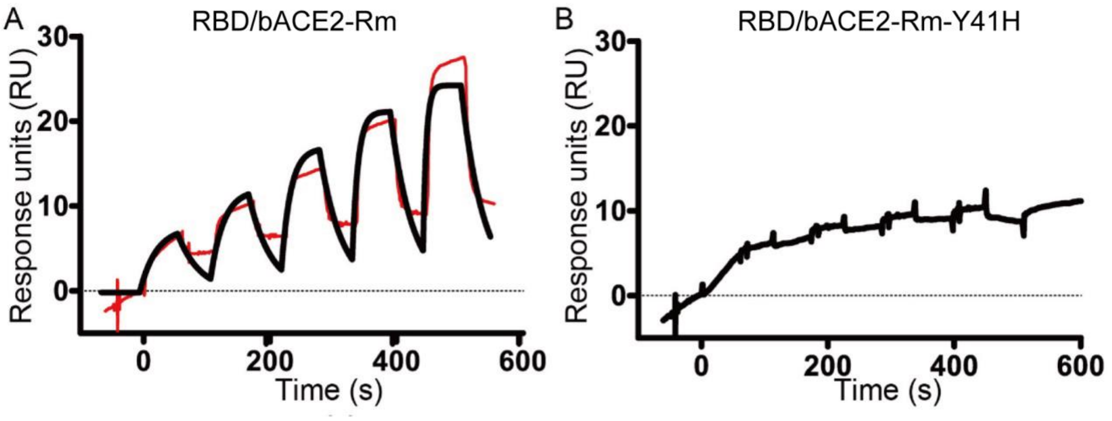


**SI Appendix, Fig. S3. SPR analysis of the binding between bACE2-Rm-Y41H mutant and bACE2-Rm-wt.** SPR assay characterisation of the binding of wild type bACE2-Rm-wt (A) or bACE2-Rm-Y41H mutant with SARS-COV-2 virus RBD. No substantial binding was observed with bACE2-Rm-Y41H mutant. The fits of the binding curves are shown in red.

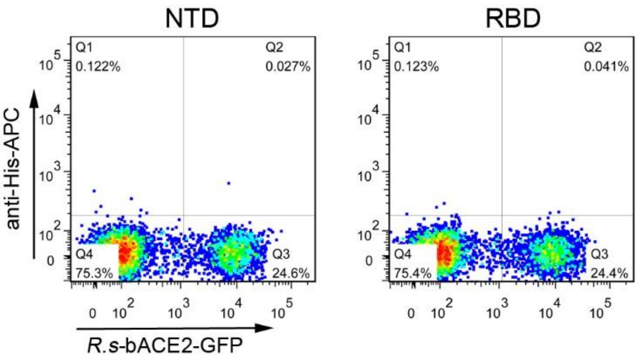


**SI Appendix, Fig. S4. Flow cytometry based assay characterizing the binding of the SARS-CoV-2 RBD with the bACE2 from *Rhinolophus sinicus* (R.s-bACE2).**

**SI Appendix, Table. S1. Binding profiles of SARS-CoV-2-RBD and SARS-CoV-RBD binding to different ACE2s**

| Interaction pairs | Ka[1](1/Ms) | Kd[2](1/s) | KD(M) |
|---|---|---|---|
| SARS-COV-2-RBD/bACE2-Rm-E.coli | $1.02\times10^{5}$ | $1.32\times10^{-1}$ | $1.30\times10^{-6}$ |
| SARS-COV-2-RBD/bACE2-Rm-insect | $4.61\times10^{4}$ | $6.92\times10^{-2}$ | $1.50\times10^{-6}$ |
| SARS-COV-2-RBD/bACE2-Rm-293T cell | $4.93\times10^{5}$ | $1.37\times10^{-1}$ | $2.78\times10^{-6}$ |
| SARS-CoV-RBD/bACE2-Ra | - | - | - |
| SARS-COV-2-RBD/hACE2 | $2.93\times10^{5}$ | $5.97\times10^{-1}$ | $2.04\times10^{-8}$ |

[1] ka, association rate constant.

[2] kd, dissociation rate constant.

**SI Appendix, Table. S2. Crystallographic data collection and refinement statistics**

| | bACE2-Rm/SARS-CoV-2-RBD |
|---|---|
| **Data collection** | |
| Space group | I422 |
| Wavelength (Å) | 0.97853 |
| Unit cell dimensions | |
| a, b, c (Å) | 163.20, 163.20, 211.67 |
| α, β, γ (°) | 90, 90, 90 |
| Resolution (Å) | 50-3.2 (3.31-3.20)* |
| Unique reflections | 24258 (2387) |
| $R_{merge}$ | 0.163/1.790 |
| $I/\sigma$ | 6 (4.3) |
| Completeness (%) | 99.9 (100) |
| Redundancy | 13.4 (13.5) |
| **Refinement** | |
| Resolution (Å) | 48.1-3.184 |
| $R_{work}$ / $R_{free}$ | 0.2185/0.2708 |
| No. atoms | |
| Protein | 7316 |
| Ligands | 1 |
| Water | 0 |
| **R.m.s. deviations** | |
| Bond lengths (Å) | 0.002 |
| Bond angles (°) | 0.682 |
| **Ramachandran plot** | |
| Favored (%) | 99.11 |
| Allowed (%) | 0.89 |

| | |
|---|---|
| Outliers (%) | 0.00 |

*Values in parentheses are for highest-resolution shell.

**SI Appendix, Table. S3. Cryo-EM data collection, refinement and validation statistics of bACE2**

| | **bACE2-Rm** |
|---|---|
| **Data collection and processing** | |
| Magnification | 130K |
| Voltage (kV) | 300 |
| Electron exposure ($e^-$/ $Å^2$) | 50 |
| Defocus range (μm) | -1.8 ~ -2.2 |
| Pixel size (Å) | 0.99375 |
| Symmetry imposed | C1 |
| Final particle images (no.) | 62,289 |
| Map resolution (Å) | 3.2 |
| FSC threshold | 0.143 |
| **Refinement** | |
| Initial model used (PDB code) | 6LZG |
| Model resolution range (Å) | up to 3.2 |
| FSC average (model to map) | |
| Whole unit cell | 0.7702 |
| Around atoms | 0.7738 |
| Model composition | |
| Non-hydrogen atoms | 4853 |
| Protein residues | 4852 |
| Ligands | 1 |
| *B* factors ($Å^2$) | |
| Protein | 70.0 |
| Ligand | 76.5 |
| R.m.s. deviations | |
| Bond lengths (Å) | 0.002 |
| Bond angles (°) | 0.418 |
| Validation | |
| MolProbity score | 1.45 |
| Clashscore | 4.88 |
| Poor rotamers (%) | 0.00 |
| Ramachandran plot | |
| Favored (%) | 96.80 |
| Allowed (%) | 3.20 |
| Outliers (%) | 0.00 |

**SI Appendix, Table. S4. Comparison of SARS-CoV-2-RBD binding to hACE2 and bACE2-Rm**

| SARS-CoV-2-RBD | humanACE2 | bACE2-Rm |
|---|---|---|
| **K417** | D30 (4,1) | D30 (6,1) |
| **G446** | Q42 (4, 1) | E42 (4) |
| **Y449** | D38 (9, 1), Q42 (4, 1) | D38 (9, 1), E42 (8, 1) |
| **Y453** | H34 (5, 1) | S34 (3) |
| **L455** | D30 (2), K31 (2), H34 (9) | D30 (7), K31 (3), S34 (2) |
| **F456** | T27 (5), D30 (4), K31 (5) | K27 (11), D30 (4), K31 (4) |
| **Y473** | T27 (1) | K27 (6, 1) |
| **A475** | S19 (3, 1), Q24 (4),T27 (2) | E24 (8, 1), K27 (2) |
| **G476** | S19 (4), Q24 (5) | E24 (9) |
| **E484** | K31 (1) | K31 (2, 1) |
| **F486** | L79 (2), M82 (9), Y83 (11) | L79 (2), N82 (7), Y83 (8) |
| **N487** | Q24 (15, 1), Y83 (8, 1) | E24 (16, 1), Y83 (5, 1) |
| **Y489** | T27 (7), F28 (7), K31 (6), Y83 (1) | K31 (9), F28 (10), Y83 (1, 1), K27 (4), E24 (1) |
| **F490** | K31 (2) | K31(3) |
| **L492** | | |
| **Q493** | K31 (3), H34 (6), E35 (8) | K31 (7), S34 (6), K35 (3) |
| **G496** | D38 (5), K353 (7, 1) | D38 (4), K353 (6, 1) |
| **Q498** | D38 (1), Y41 (8), Q42 (8, 3), L45 (3) | Y41 (7), D38 (4), E42(3), K353 (2) |
| **P499** | | K330 (1) |
| **T500** | Y41 (7, 1), L45 (1), N330 (8), D355 (8, 1), R357 (3) | K330 (11, 1), Y41 (10, 1), L45 (3), D355 (9), R357 (3) |
| **N501** | Y41 (8, 1), K353 (11) | Y41 (14, 1), K330 (1), K353 (11) |
| **G502** | K353 (4, 1), G354 (7), D355 (1) | K353 (6, 1), G354 (6), D355 (1) |
| **Y505** | E37 (7), K353 (28), G354 (4), R393 (1) | K353 (27), E37 (8, 1) , R393 (2), G354 (2) |
| **Total** | 288, 16 | 311,14 |

The numbers in parentheses of hACE2 and bACE2-Rm residues represent the number of vdw contacts between the indicated residues with SARS-CoV-2-RBD. The numbers with underline suggest numbers of potential H-bonds between the pairs of residues. wdw contact was analyzed at a cutoff of 4.5 Å and H-bonds at a cutoff of 3.5 Å.

## SI Appendix, Table. S5. Conservation analysis of the residues involved in ACE2 binding to SARS-CoV-2-RBD among 31 bats

| | 24 | 27 | 28 | 30 | 31 | 34 | 35 | 37 | 38 | 41 | 42 | 45 | 79 | 82 | 83 | 330 | 353 | 354 | 355 | 357 | 393 | Substitutions |
|---|---|---|---|---|---|---|---|---|---|---|---|---|---|---|---|---|---|---|---|---|---|---|
| ADN93471\|Rhinolophus_macrotis | E | K | F | D | K | S | K | E | D | Y | E | L | L | N | Y | K | K | G | D | R | R | |
| QMQ39222.1\|Rhinolophus affinis | R | I | F | D | N | R | E | E | E | Y | Q | L | L | N | Y | N | K | G | D | R | R | 7 |
| XP_023609439\|Myotis_lucifugus | K | I | F | E | N | S | K | E | D | H | E | L | L | T | Y | N | K | G | D | R | R | 7 |
| ADJ19219\|Rousettus_leschenaultii | L | T | F | E | K | T | E | E | D | Y | Q | L | L | T | Y | K | K | G | D | R | R | 7 |
| BAH02663\|Rhinolophus_ferrumequinum | L | K | F | D | D | S | E | E | N | H | Q | L | L | N | F | N | K | G | D | R | R | 8 |
| ADN93472\|Rhinolophus_sinicus | E | I | F | D | K | T | K | E | D | H | Q | L | L | N | Y | N | K | G | D | R | R | 5 |
| ADN93477\|Rhinolophus_pusillus | K | K | F | N | D | S | E | E | D | Y | Q | L | L | N | Y | N | K | G | D | R | R | 6 |
| ACM45790\|Rhinolophus_ferrumequinum | L | T | F | E | K | T | E | E | D | Y | Q | L | L | T | Y | K | K | G | D | R | R | 7 |
| BAF50705\|Rousettus_leschenaultii | L | T | F | E | K | T | E | E | D | Y | Q | L | L | T | Y | K | K | G | D | R | R | 7 |
| XP_015974412\|Rousettus_aegyptiacus | L | T | F | E | K | T | E | E | D | Y | Q | L | L | T | Y | K | K | G | D | R | R | 7 |
| XP_011361275\|Pteropus_vampyrus | L | T | F | E | K | T | E | E | D | Y | Q | L | L | A | Y | K | K | G | D | R | K | 7 |
| XP_006911709\|Pteropus_alecto | L | T | F | E | K | T | E | E | D | Y | Q | L | L | A | Y | K | K | G | D | R | K | 7 |
| XP_019522936\|Hipposideros_armiger | L | E | F | D | K | T | E | E | D | H | L | L | R | D | Y | N | K | G | D | R | R | 8 |
| ADN93470\|Rhinolophus_ferrumequinum | L | K | F | D | D | S | E | E | N | H | Q | L | L | N | F | N | K | G | D | R | R | 8 |
| ALJ94034\|Rhinolophus_landeri | L | T | F | D | D | S | A | E | N | Y | Q | L | H | N | F | N | K | G | D | R | R | 9 |
| ALJ94035\|Rhinolophus_alcyone | L | I | F | D | N | S | E | E | N | H | Q | L | H | N | F | N | K | G | D | R | R | 10 |
| ABU54053\|Rhinolophus_pearsonii | R | T | F | D | K | H | E | E | D | H | Q | L | L | D | Y | N | K | D | D | R | R | 9 |
| ACT66275\|Rhinolophus_sinicus | L | I | F | D | E | S | E | E | N | Y | Q | L | L | N | Y | N | K | G | D | R | R | 7 |
| ADN93475\|Rhinolophus_sinicus | R | T | F | D | E | S | E | E | N | Y | Q | L | L | N | Y | N | K | G | D | R | R | 7 |
| AGZ48803\|Rhinolophus_sinicus | E | M | F | D | K | T | K | E | D | H | Q | L | L | N | Y | N | K | K | D | R | R | 6 |
| XP_028378317\|Phyllostomus_discolor | D | K | F | E | N | N | E | E | E | Y | Q | L | L | N | Y | N | K | K | D | R | R | 9 |
| XP_024425698\|Desmodus_rotundus | E | T | F | E | N | T | E | E | E | Y | Q | L | I | T | Y | N | N | G | D | R | R | 11 |
| XP_016058453\|Miniopterus_natalensis | K | K | F | E | G | S | Q | E | D | F | E | L | L | I | Y | N | K | N | D | R | R | 8 |
| ACT66266\|Pipistrellus_abramus | E | R | F | V | K | H | E | E | N | H | E | L | I | G | Y | D | K | N | D | R | R | 10 |

| | | | | | | | | | | | | | | | | | | | | | | |
|---|---|---|---|---|---|---|---|---|---|---|---|---|---|---|---|---|---|---|---|---|---|---|
| XP_027986092\|Eptesicus_fuscus | N | I | F | E | N | S | E | E | D | H | E | L | L | T | F | N | K | N | D | R | R | 10 |
| XP_008153150\|Eptesicus_fuscus | N | I | F | E | N | S | E | E | D | H | E | L | L | T | Y | N | K | G | D | R | R | 7 |
| XP_015426919\|Myotis_davidii | K | I | F | D | N | S | K | E | D | H | E | L | L | T | Y | N | K | G | D | R | R | 6 |
| XP_006775273\|Myotis_davidii | K | I | F | D | N | S | K | E | D | H | E | L | L | T | Y | N | K | G | D | R | R | 6 |
| XP_023609437\|Myotis_lucifugus | K | I | F | E | N | S | K | E | D | H | E | L | L | T | Y | N | K | G | D | R | R | 7 |
| XP_014399783\|Myotis_brandtii | K | I | F | E | N | S | K | E | D | H | E | L | L | T | Y | N | K | G | D | R | R | 7 |
| XP_014399782\|Myotis_brandtii | K | I | F | E | N | S | K | E | D | H | E | L | L | T | Y | N | K | G | D | R | R | 7 |
| XP_014399780\|Myotis_brandtii | K | I | F | E | N | S | K | E | D | H | E | L | L | T | Y | N | K | G | D | R | R | 7 |

Red letters indicate the substitutions in the ACE2 of 31 bats.
